# Genomic prediction offers the most effective marker assisted breeding approach for ability to prevent arsenic accumulation in rice grains

**DOI:** 10.1371/journal.pone.0217516

**Published:** 2019-06-13

**Authors:** Julien Frouin, Axel Labeyrie, Arnaud Boisnard, Gian Attilio Sacchi, Nourollah Ahmadi

**Affiliations:** 1 CIRAD, UMR AGAP, Montpellier, France; 2 AGAP, Univ Montpellier, CIRAD, INRA, Montpellier SupAgro, Montpellier, France; 3 Centre Français du Riz, Mas du Sonnailler, Arles, France; 4 Università degli Studi di Milano, Milano, Italy; International Rice Research Institute, PHILIPPINES

## Abstract

The high concentration of arsenic (*As*) in rice grains, in a large proportion of the rice growing areas, is a critical issue. This study explores the feasibility of conventional (QTL-based) marker-assisted selection and genomic selection to improve the ability of rice to prevent *As* uptake and accumulation in the edible grains. A *japonica* diversity panel (RP) of 228 accessions phenotyped for *As* concentration in the flag leaf (FL-*As*) and in the dehulled grain (CG-*As*), and genotyped at 22,370 SNP loci, was used to map QTLs by association analysis (GWAS) and to train genomic prediction models. Similar phenotypic and genotypic data from 95 advanced breeding lines (VP) with *japonica* genetic backgrounds, was used to validate related QTLs mapped in the RP through GWAS and to evaluate the predictive ability of across populations (RP-VP) genomic estimate of breeding value (GEBV) for *As* exclusion. Several QTLs for FL-*As* and CG-*As* with a low-medium individual effect were detected in the RP, of which some colocalized with known QTLs and candidate genes. However, less than 10% of those QTLs could be validated in the VP without loosening colocalization parameters. Conversely, the average predictive ability of across populations GEBV was rather high, 0.43 for FL-*As* and 0.48 for CG-*As*, ensuring genetic gains per time unit close to phenotypic selection. The implications of the limited robustness of the GWAS results and the rather high predictive ability of genomic prediction are discussed for breeding rice for significantly low arsenic uptake and accumulation in the edible grains.

## Introduction

Presence of high concentration of arsenic (*As*) in the paddy fields or in the irrigation water (>20 mg Kg^-1^ and 0.10 mg Kg^-1^, respectively) has been reported in more than 70 countries in Asia, America and Europe [1–3]. The problem, often of geological origin, affects several hundred million peoples living in the area, especially in Asia [1, 3, 4] as it translates into rice grain. Local and regional surveys have revealed tight correlation between *As* concentration in the cultivated soil, or in the irrigation water, and in the rice plant [2, 5]. *As* accumulation in the rice plant is the highest in the roots, followed by the straw, the whole (cargo) grain and the polished (white) grain [2, 5, 6]. Presence of *As* in the paddy field also affects crop growth and development and, consequently, crop yield [6].

Alternate wetting and drying of the paddy field during the cropping season is the most effective way to achieve agronomic mitigation. In aerobic soils, the oxidative immobilization of *As* reduces its phyto-availability and uptake in rice [7]. Application of silicon fertilizer can also reduce the concentration of *As* in the rice plant [8]. A second category of mitigation options relies on rice genetic improvement to reduce *As* uptake and/or its translocation from the vegetative organs to the edible grains. A third category of mitigation relies on rice cooking methods. Washing and/or cooking in excess water reduce rice grains’ *As* content [9, 10].

Mechanisms of rice plant response to soil *As* excess have been reported to be similar to those observed for other types of soil chemical toxicity such as iron and salt [11]. However, the exact mechanisms related to the phytotoxic effects of *As*, and the rice defense response against *As* remain poorly understood. In aerobic cultivation, the predominant form of soil *As* is arsenate, *As*(OH)_5_ or *As*(V), and its uptake by plants involves phosphate transporters [12]. Overexposure to arsenate triggers reduced expression of genes coding for arsenate/phosphate transporters such as PHT1 [13]. At the same time, the arsenate taken up undergoes chemical reduction to a more highly toxic species, arsenite [*As*(III)] [14, 15]. The arsenite is then either excreted into the rhizosphere [16, 17], or transported to aboveground organs [18], and/or detoxified by complexation as phyto-chelatines and compartmentalized in the cell vacuoles [19]. Within the anaerobic conditions of paddy fields, the predominant form of *As* is arsenite [20]. Arsenite enters root cells through aquaporin type membrane ports [21]. Transporters involved in this process include silicon transporters Lsi1 (influx) and Lsi2 (efflux) [21, 22] and several silicon-independent pathways [23, 24].

Significant genetic diversity for *As* accumulation has been reported in rice, under overexposure to *As* in hydroponic cultivation and in field experiments [25–28]. Analysis of grain *As* content in 421 rice accessions grown in six sites distributed in Bangladesh, China and USA revealed from 3 to 34 fold variation in each site [29]. It also revealed that accessions belonging to the *Aus* genetic group, originated from the Indian subcontinent, had the highest *As* contents.

Using recombinant inbred lines (RIL) from bi-parental crosses, several QTLs involved in *As* accumulation have been mapped [25, 30, 31]. Likewise, GWAS with the phenotypic data produced in the above described multilocation trial, detected several significant associations for grain *As* content [29, 32]. However, none of the significant associations mapped in the vicinity (distance of less than 200 kb) of the Os02g51110 and Os03g01700 loci coding for Lsi1 and Lsi2 proteins [21, 22], that can play a central role in rice response to *As* overexposure. Likewise, very few significant associations detected by GWAS, colocalized with QTLs mapped in RIL populations [32]. Analysis of *As*-induced genome-wide modulation of transcriptomes of rice seedling roots revealed up-regulation of several hundred genes, confirming the complexity of the gene network involved in response to *As* overexposure [33–36]. Gene families with differential gene expression in *As* tolerant and *As*-susceptible genotypes include glutathione S-transferases, cytochrome P450s, heat shock proteins, metal-binding proteins, and a large number of transporters and transcriptions factors such as MYBs [37]. More recently, using a reverse genetics approach, [38] showed that OsHAC1;1 and OsHAC1;2 functioned as arsenate reductases and played a role in the control of *As* accumulation in rice. Similar crucial role in *As* accumulation in rice was reported for OsHAC4 [16]. Based on these findings, some authors recently advocated turning to gene-editing technology for the control of *As* accumulation in rice grains [7, 24].

Genomic selection (GS) has recently emerged as an alternative option to conventional marker-assisted selection targeting mapped QTLs [39–41]. By shifting the plant breeding paradigm from “breeding by design” to a “genome-wide-approach”, GS provides genomic estimated breeding values (GEBV) based on all available marker data, instead of focusing on a limited set of markers that tag putative genes or QTLs. In the last few years, successful proof of GS concept has been reported in maize [42], wheat [43], barley [44] and oats [45]. In rice, moderate to high predictive ability (PA) of GEBV has been reported for a variety of quantitative traits in experiments with the progeny of bi-parental crosses and in diverse germplasm collections [46–51]. In particular, it was shown that rice diversity panels provide accurate genomic predictions for complex traits in the progenies of biparental crosses involving members of the panel [52]. It was also shown that genomic prediction accounting for genotype by environment interaction offered an effective framework for breeding simultaneously for adaptation to an abiotic stress and for performance under normal cropping conditions in rice [53].

The above reported findings suggest that *As* uptake and accumulation in rice grains are a complex process involving a large number of QTLs. We thus, hypthesized that genomic prediction offers the most effective marker assisted breeding approach for ability to prevent *As* accumulation in rice grains. To test this hypothesis the potential of two marker assisted selection approaches to improve the ability of rice to restrict *As* accumulation in the grains was evaluated. First, field phenotypic data (leaf and grain *As* content of rice plants grown in paddy fields with rather high *As* concentrations) and genotypic data from a reference diversity panel, were used to either map QTLs involved in *As* accumulation through GWAS or to train genomic prediction models. Second, using similar phenotypic and genotypic data from a panel of advanced lines from a breeding program, congruence between GWAS results in the two populations was analyzed, and the predictive ability of genomic prediction across the two populations was evaluated. The results identified genomic prediction as the most promising approach to improve the ability of rice to restrict *As* uptake and its accumulation in the grains.

## Methods

### Plant material

The plant material phenotyped comprised a diversity panel of 300 accessions and a set of 100 advanced inbred lines (F5, F6 or F7), all belonging to the *japonica* subspecies of *O*. *sativa*. The diversity panel, hereafter referred to as the reference population (RP), was composed of 214 accessions representing the European Rice Core Collection [54], and 86 accessions of direct interest for the Camargue-France breeding program (S1 Table). The 100 advanced breeding lines hereafter referred to as the validation population (VP), was composed of elite lines of the rice breeding program run by the *Centre Français du Riz* (CFR) and Cirad, in the Camargue region, France. Within this plant material, 228 accessions of the RP and 95 lines of the VP were genotyped and served for GWAS and genomic prediction studies.

### Field trials and phenotyping

Field trials were conducted at the CFR experimental station, Mas d’Adrien (43°42’13.77”N; 4°33’44.71”E; 3 m asl.), under a standard irrigated rice cropping system. The RP was phenotyped in two consecutive years (2014 and 2015), the VP only in 2016. In 2014, the 300 accessions of RP were phenotyped under an augmented randomized complete block design repeated twice, each block being composed of 25 tested accessions and two check varieties (Albaron and Brio). To confirm the results of the 2014 trial, in 2015, 50 accessions of RP, with contrasted *As* content performances in 2014, were phenotyped in a complete randomized blocks design with eight replicates. In both 2014 and 2015 trials, the size of the individual plot was one row of 15 plants. In 2016, each of the 100 advanced lines of VP was represented by five full-sib lines and the size of the individual plot for each full-sib line was one row of 15 plants.

In each field trial, the concentration of total *As* in the flag leaf (FL-*As*) and in the cargo grain (CG-*As*) was measured and the CG-*As*/FL-*As* ratio (Ratio) calculated. In the 2014 and 2015, three biological samples were prepared for each individual plot to measure FL-*As*. Each biological sample was composed of three flag leaves of three different plants. Each biological sample was oven-dried at 75°C for 120 h, ground, mineralized, and total *As* concentration was measured using the inductively coupled plasma mass spectrum (ICP-MA; Bruker Aurora ICP Mass Spectrometer). For each biological sample, total *As* was measured in at least two technical samples and averaged to establish the sample phenotype. Data from the three biological samples were averaged to establish the plot phenotype. A similar procedure was applied to CG-*As* measurement in which the biological samples were composed of three panicles. These panicles were threshed after oven drying, the paddy grains were de-husked, and the resulting cargo grains were ground before undergoing the mineralization procedure.

In 2016, FL-*As* and CG-*As* were measured in one randomly chosen sib-line in each advanced line. Two biological samples were prepared from each chosen sib-line: one biological sample from an individual plant that was also used for DNA extraction and genotyping (see below), and a second sample from the bulk of at least three plants.

In each field trial, the soil total *As* content was measured before sowing and after harvest. Likewise, in each field trial, total *As* content of irrigation water was monitored once a month during the rice cropping cycle. Soil samples were collected from 10–15 cm depth in 1 m^2^ area, in three points of the field. Water samples were collected monthly from the irrigation canal at its junction point with the experimental field. The total *As* concentration was measured using the ICP-AES (Inductively coupled plasma atomic emission spectrometry) method.

### Genotypic data

Genotypic data were produced by two distinct genotyping by sequencing (GBS) experiments, for 228 accessions of RP and 95 lines of VP (because of limited funds available). In both cases, DNA libraries were prepared at the Regional Genotyping Technology Platform (http://www.gptr-lr-genotypage.com) hosted at Cirad, Montpellier, France. For each accession, genomic DNA was extracted from the leaf tissues of a single plant, using the MATAB method [55]. Each DNA sample was diluted to 100 ng/μl and digested separately with the restriction enzyme *ApekI*. DNA libraries were then single-end sequenced in a single-flow cell channel (i.e. 96-plex sequencing) using an Illumina HiSeq2000 (Illumina, Inc.) at the Regional Genotyping Platform (http://get.genotoul.fr/) hosted at INRA, Toulouse, France. The fastq sequences were aligned to the rice reference genome, Os-Nipponbare-Reference-IRGSP-1.0 [56] with Bowtie2 (default parameters). Non-aligning sequences and sequences with multiple positions were discarded. Single nucleotide polymorphism (SNP) calling was performed using the Tassel GBS pipeline v5.2.29 [57]. The initial filters applied were the quality score (>20), call rate >80% the count of minor alleles (>1), and the bi-allelic status of SNPs. In the second step, loci with minor allele frequency (MAF) below 2.5% and with more than 20% missing data were discarded. The missing data were imputed using Beagle v4.0 [58].

### Analysis of phenotypic data

In 2014, plot level phenotypic data of the 300 accessions of RP were modeled for each trait as:

$$Y_{ijk}=\mu +a_{i}+r_{j}+b_{jk}+{\beta (r)}_{jk}+{\left(ar\right)}_{ij}+e_{ij}$$

where *Y*_*ijk*_ is the observed phenotype of accession *i* in replicate *j* and bloc *k*, μ is the overall mean, *a*_*i*_ the accession effect, *r*_*j*_ the replicate effect, *b*_*jk*_ the check effect considered as quantitative covariate, *β*(*r*)_*jk*_ the block effect within the replicate, (*ar*)_*ij*_ the interaction between accessions and replicates, and *e*_*ij*_ the residual.

In 2015, plot level phenotypic data of the 50 accessions of RP were modeled for each trait as *Y*_*ij*_ = μ+*a*_*i*_+*r*_*j*_+(*ar*)_*ij*_+*e*_*ij*_ where *Y*_*ij*_ is the observed phenotype of accession *i* in bloc *j*, μ is the overall mean, *a*_*i*_ the accession effect, *r*_*i*_ the replicate effect, (*ar*)_*ij*_ the interaction between accession *i* and replicate *j*, considered as random, and *e*_*ij*_ the residual. For each dataset and each trait, least square means were estimated using the mixed model procedure of Minitab 18.1.0 statistical software.

Broad-sense heritability was calculated for each trait as: $h^{2}=\sigma_{g}^{2}/(\sigma_{g}^{2}+\sigma_{e}^{2}/n)$, where $\sigma_{g}^{2}$ and $\sigma_{e}^{2}$ are the estimates of genetic and residual variances, respectively, derived from the expected mean squares of the analysis of variance and *n* is the number of replicates. The computed CG-*As*/FL*-As* ratio were subjected to the angular transformation *2Arcsin square root* before analysis.

### Genotypic characterization of RP and VP

The genetic structure of 228 accessions of RP and 95 advanced lines of VP was analyzed jointly using a distance-based method [59]. First, a matrix of 3,620 SNPs was extracted from the working genotypic dataset of 22,370 SNPs common to RP and VP, by discarding loci that had imputed data and by imposing a minimum distance of 25 kb between two adjacent loci. Then, an unweighted neighbor-joining tree based on dissymmetry matrix was constructed using DarWin v6 software [59].

The speed of decay of linkage disequilibrium (LD) in RP and VP was estimated by computing r^2^ between pairs of markers on a chromosome basis using Tassel 5.2 software [60], and then averaging the results by distance classes using XLStat.7.

### Association analysis

Separate association analyses were performed with phenotypic and genotypic data from 228 accessions of RP and from 95 advanced lines of VP. A single marker regression-based association analysis was performed for each phenotypic trait under a mixed linear model (MLM), in which marker and population structure (Q matrix) effects were considered as fixed and the kinship effect (K matrix) was considered as random. The MLM was run under the exact method option of Tassel 5.2 software [60], where the additive genetic and residual variance components are re-estimated for each SNP. For each SNP tested, Tassel 5.2 computed a p-value, the log likelihood of the null and alternative models, and the fixed-effect weight of the SNP with its standard error. The threshold to declare the association of a SNP marker with a trait to be significant was set at a probability level of 1e-05. Genes underlying the significant loci were analyzed using the MSU database (http://rice.plantbiology.msu.edu/) search and gene annotation.

### Genomic prediction

#### Construction of the incidence matrix

In order to reduce possible negative effects of redundancy of marker information on the predictive ability of genomic predictions and to reduce computing time, redundant SNPs were discarded as follows. First, using the genotypic dataset of the RP (N = 228 entries and P = 22,370 SNPs), for each SNP pairwise LD with all other SNPs was calculated. Second, among each group of SNPs in complete LD (r^2^ = 1), the first SNP along the chromosome was maintained and all the others were discarded. This procedure reduced the total number of SNP loci to 16,902. Once the list of these SNPs was established, the incidence matrix of 16,902 SNP was constructed for the VP accordingly.

#### Cross-validation experiment in the RP

Three whole-genome regression models, the genomic best linear unbiased prediction (GBLUP), the Bayesian regression BayesA, and the reproducing kernel Hilbert spaces regressions (RKHS) were used to regress the FL-*As*, CG-*As* and Ratio traits of RP accessions on their genotype at 16,902 SNP loci. GBLUP is a parametric model (i.e. genetic values are approximated by using linear regression procedures) with Gaussian prior density of marker effects, that hypothesizes homogenous marker effects and strictly additive determinism of the genetic effects [61]. It was implemented using the genomic matrix G = M*M’ (M being the incidence matrix). BayesA is a parametric model with a scaled-t prior density of marker effects, i.e. density with higher mass at the neighborhood of zero, inducing strong shrinkage toward zero of effects of markers with small effects [62]. It hypothesizes that some markers are linked to QTL while others are in regions that do not harbor QTL. RKHS is a semiparametric regression model that combines the additive genetic model with a kernel function and has the ability to capture more complex genetic determinisms [63]. The three models were implemented using the *BGLR* statistical package [64]. The default parameters for prior specification were used and the number of iterations for the Markov chain Monte Carlo (MCMC) algorithm was set to 25,000 with a burn-in period of 5,000.

The cross-validation experiments used 171 (3/4) of the 228 accessions of the RP as the training set and the remaining 57 (1/4) accessions as the validation set. Each cross-validation experiment was repeated 100 times using 100 independent partitioning of the RP into training set and validation set. For each independent partitioning, the correlation between the predicted and the observed phenotype was calculated so as to obtain 100 correlations for each cross-validation experiment. The predictive ability of each cross-validation experiment was computed as the mean value of the 100 correlations.

To analyze sources of variation in the predictive ability of genomic predictions, the correlation (*r*) of repetition was transformed into a Z statistic using the equation: *Z* = 0.5 {*ln*[1+*r*]−*ln*[1−*r*]} and analyzed as a dependent variable in an analysis of variance. After estimation of confidence limits and means for Z, these were transformed back to *r* variables.

#### Genomic prediction across populations

The predictive ability of genomic prediction across populations was evaluated under three scenarios of composition of the training set. Under the first scenario (S1), the training set included all 228 accessions of RP. Under S2, the training set was composed of 100 accessions of the RP with the lowest average pairwise Euclidian distances with the 95 lines of the VP. Under S3, 100 accessions of the training set were selected among the 228 accessions of RP, using the CDmean method of optimization of the training set [65]. In this 3rd scenario, a dedicated training set was selected for each phenotypic trait to account for trait heritability. Predictions were performed with the three above-described statistical methods (GBLUP, BayesA and RKHS) using the *BGLR* statistical package [64]. For each trait (FL-*As*, CG-*As*, and CG-*As*/FL-*As* Ratio), the predictive ability of the prediction experiment was calculated as the correlation between the predicted and the observed phenotypes of the 95 lines.

## Results

### Phenotypic diversity for arsenic content

In 2014, soil analyses before crop establishment and after crop harvest revealed similar *As* concentrations of about 10 mg kg^-1^ dry soil weight. During the same period, the monthly survey of the irrigation water revealed variable *As* contents (0.014 to 0.034 mg l^-1^) with an average of 0.021 mg l^-1^. Similar soil and water *As* contents were observed in 2015 and 2016 (S2 Table).

#### Variation in arsenic content in the reference population

**Fig 1:**
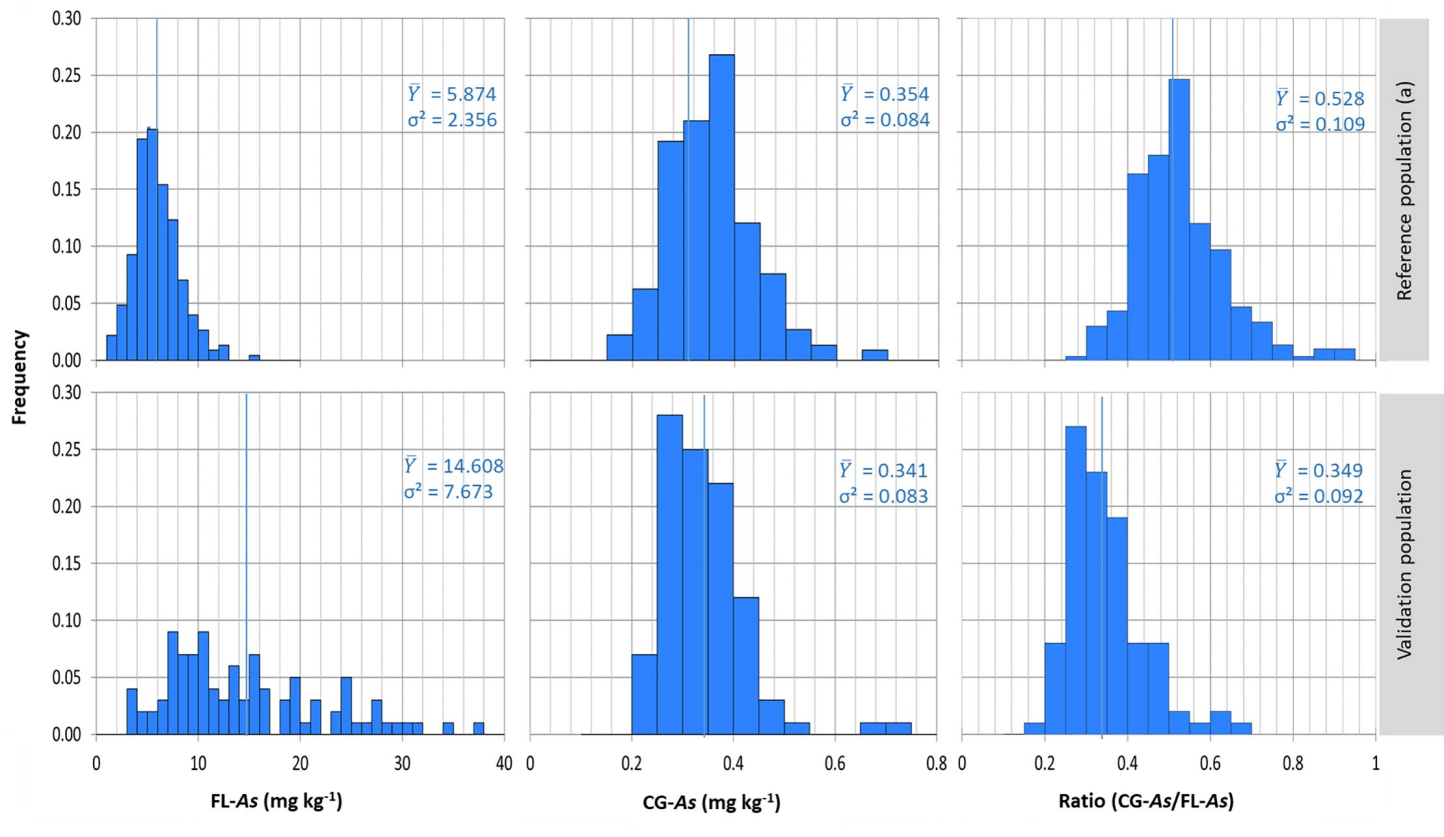
Distribution of adjusted phenotypic values for flag leaf arsenic content (FL-*As*), Cargo grain arsenic content (CG-*As*), and the CG-*As*/FL-*As* ratio, in the reference (RP) and validation (VP) populations. a: data from 2014 field experiment. The vertical blue line indicates the mean value of arsenic content the in the population.

The distributions of the three traits (FL-*As*, CG-*As* and Ratio) measured in 2014 among the 300 accessions of RP are presented in Fig 1. Variation in FL-*As* ranged from 1.393 (Bexibell accession) to 15.509 mg kg^-1^ (Auzgusta accessions) and averaged 5.881 mg kg^-1^ of dry weight. Variation in CG-*As* ranged from 0.147 (Suweon accession) to 0.656 mg kg^-1^ (Octet accession) and averaged 0.335 mg kg^-1^ (S1 Table). Partitioning of the observed phenotypic variations into different sources of variation via the mixed model analysis revealed a highly significant effect of accession for the three traits considered (Table 1). The model R^2^ was greater than 0.701 for the three traits, indicating a good fit of the model. Broad-sense heritability tended to confirm this trend, with values ranging from 0.803 to 0.861 in 2014 (Table 1). The determination coefficient between FL-*As* and CG-*As* was rather low but highly significant (R^2^ = 0.204, p < 0.0001). This rather loose relationship between FL-*As* and CG-*As* corroborates the significant accession effect observed for the Ratio trait.

**Table 1:** Variance components of three phenotypic traits in the reference population (RP) evaluated in 2014 and in 50 selected accessions of RP evaluated in 2015.

Trial	Factors	FL-*As*		CG-*As*		Ratio	
300 RP accessions 2014	Accession (A)	10.389	***	0.012	***	0.022	***
	Replicate (R)	6.681	NS	0.121	***	0.055	NS
	(A) x (R)	8.373	NS	0.005	***	0.009	NS
	Residual	4.134		0.004		0.011	
	h^2^	0.831		0.864		0.803	
50 RP accessions 2015	Accession	425.303	***	0.042	***	0.054	***
	Replicate	286.182	***	0.006	***	0.071	***
	Residual	17.448		0.002		0.005	
	h^2^ (SE)	0.995		0.994		0.911	

FL-*As*: flag leaf arsenic content; CG-*As*: cargo grain arsenic content; Ratio: CG-*As*/FL-*As*

h^2^: broad sense heritability

***: significant at p≤0.001; NS: not significant.

In 2015, the range of variation in FL-*As* among the 50 accessions of RP with contrasted *As* contents in 2014 was much larger (from 3.692 to 34.689; average of 16.833 mg kg^-1^), while the range of variation in CG-*As* was slightly narrower (0.169 to 0.493; average of 0.338 mg kg^-1^). However, these differences in the range of variation did not change the relative ranking of the 50 accessions observed in 2014. Indeed, the Spearman coefficient of rank correlation between performances of the 50 RP accessions in 2014 and 2015 was r = 0.724 (p < 0.0001) for FL-*As*, r = 0.681 (p < 0.0001) for CG-*As*, and r = 0.587 (p < 0.0001) for Ratio. The determination coefficient between FL-*As* and CG-*As* of the 50 accessions in 2015 was higher (R^2^ = 0.563, p < 0.0001) than the one observed in 2014 for the 300 accessions.

#### Variation in arsenic content in the validation population

Variation in FL-*As* among the 95 accessions in the VP ranged from 3.241 to 37.760 mg kg^-1^ and averaged 14.651 mg kg^-1^ (Fig 1). Variation in CG-*As* ranged from 0.208 to 0.729 mg kg^-1^ and averaged 0.341 mg kg^-1^. The determination coefficient between the FL-*As* and CG-*As* was low but highly significant (R^2^ = 0.20, p < 0.0001). The computed Ratio varied between 0.179 and 0.636 and averaged 0.336.

### Genetic structure of the reference and validation populations

The RP and VP genotyping experiment yielded 39,497 and 67,658 SNP loci, respectively, among which 22,370 were common to the two populations. This working dataset is presented in S3 Table. The 22,370 SNP markers of the working dataset were unevenly distributed along the 12 chromosomes (S1 Fig; S4 Table). Average marker density was one SNP every 17.1 kb. However, it ranged from one SNP every 10.7 kb in chromosome 11 to 26.7 kb in chromosome 9. The number of pairs of loci with distance greater than 250 kb, 500 kb and 1,000 kb were 175, 27 and 1, respectively.

**Fig 2:**
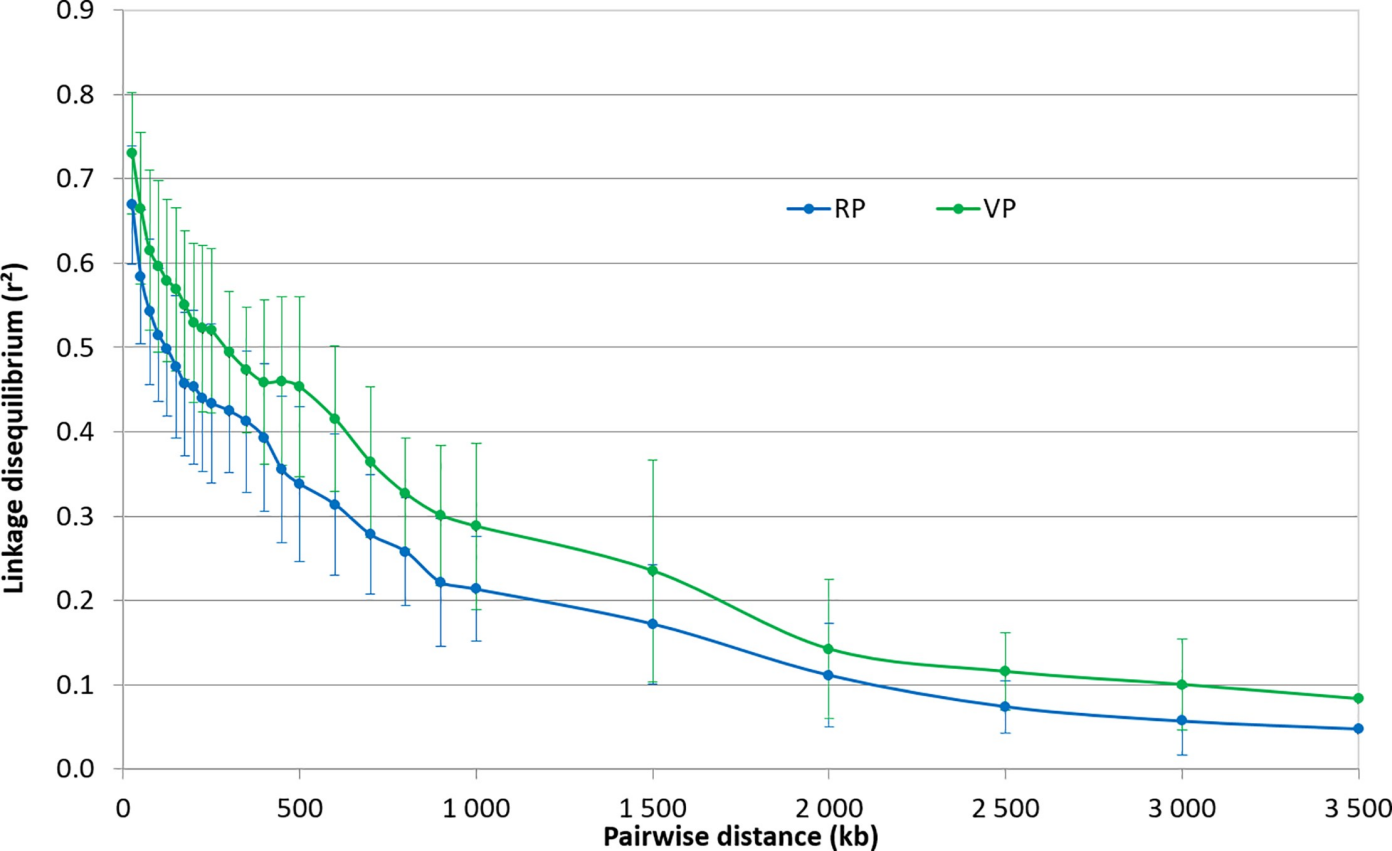
Patterns of decay in linkage disequilibrium in the reference population (RP) and in the validation population (VP). The curves represents the average r^2^ among the 12 chromosomes; the bars represent the associated standard deviation.

The decay of LD over physical distance in the two populations is presented in Fig 2. For between-marker distances of 0 to 25 kb, the average r^2^ was 0.671 and 0.733 in RP and VP, respectively. In the RP, the r^2^ value dropped to half its initial level at around 450 kb, reached 0.2 at 1.25 Mb, and 0.1 at 2.10 Mb. In the VP, r^2^ reached the 0.2 threshold only at pairwise distances of around 1.70 Mb, and the 0.1 threshold at distances above 3,0 Mb. No major difference in LD decay was observed between the 12 chromosomes. Given these extents of average LDs, marker density and distribution along the chromosome should not be a major limiting factor for the next steps of the study, i.e. detection of significant associations and genomic prediction. Both RP and VP populations showed similar MAF patterns for the 22,370 common SNP loci. RP and VP had the same minor allele in 95.4% of the common loci. In both populations, the MAF distribution was slightly skewed toward low frequencies, the average MAF was close to 22.2%, and the proportion of loci with MAF < 10% was close to 75%. Likewise, the Spearman correlation between the MAF of the 21,343 loci with identical minor alleles in the two populations was r = 0.849 (p <0.011).

**Fig 3:**
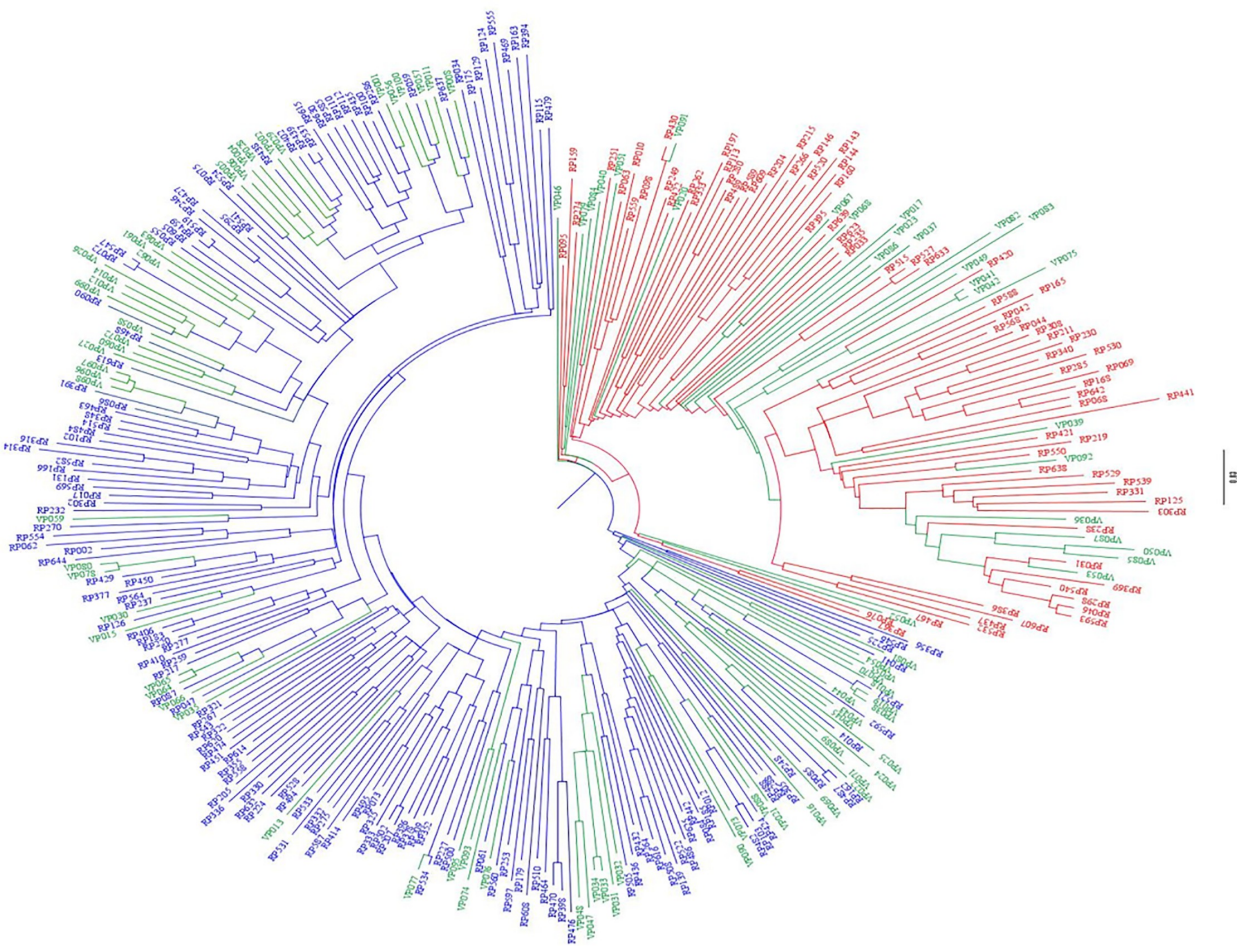
Unweighted neighbor-joining tree based on simple matching distances constructed from the genotype of 228 accessions of the reference population (RP) and 95 advanced lines of the validation population (VP), using 3,620 SNP markers. Green: VP; Red and blue: RP accessions belonging to tropical *japonica* and temperate *japonica*, respectively.

Dissymmetry-based clustering of RP accessions led to two major clusters corresponding to the temperate *japonica* (65% of accessions) and tropical *japonica* (35% of accessions) sub-groups (Fig 3). The majority of the temperate *japonica* accessions are of European origin, while the majority of the tropical *japonica* accessions originate from the American continent. The inclusion of the VP lines in the dissymmetry-based clustering did not modify the clustering into two groups. Indeed, 69% of VP lines clustered with the temperate *japonica* group and the remaining 31% with the tropical *japonica* group (Fig 3; S1 Table).

### Relationship between genotypic and phenotypic diversity

Highly significant differences in FL-*As* and CG-*As* contents were observed between the temperate *japonica* and the tropical *japonica* accessions of RP (total of 300 accessions) evaluated in 2014. The former subgroup had the highest *As* contents (S5 Table; S2 Fig). Data from the 50 RP selected accessions evaluated in 2015 and from the 95 advanced lines of VP confirmed this trend.

### Association analyses

#### Association analysis in the reference population

**Fig 4:**
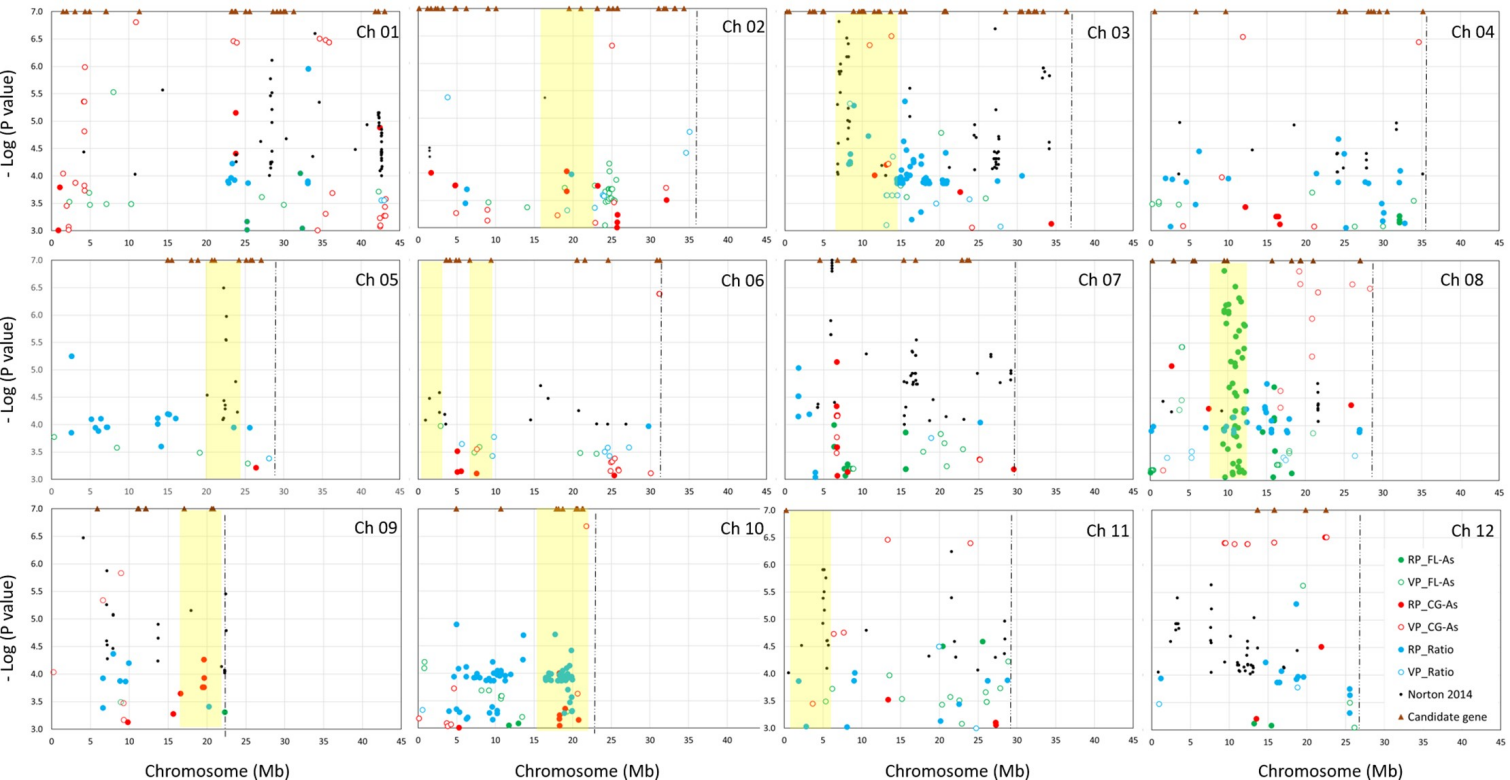
Results of association analyses in the reference population (RP) and in the validation population (VP) in the present study, and comparison with data from the literature. For the present study, data points represent SNPs significantly associated with arsenic concentration in the flag leaf (FL-*As*) in the cargo grain (CG-*As*), and the CG-*As*/FL-*As* ratio in RP and VP. Data from the literature include significant SNPs mapped by GWAS [30], QTLs for grain arsenic concentration [25, 29] and candidate genes [21, 39].

Results of association analysis for FL-*As*, CG-*As* and Ratio traits in the RP are presented in Fig 4 and in S6 Table. The number of significant associations (p-value < 1e-05) was 41 for FL-*As*, 23 for CG-*As* and 82 for Ratio. These associations represented 6, 13 and 19 independent loci, i.e. a cluster of SNPs with a distance of less than 1.25 Mb between two consecutive significant SNPs, corresponding to the average LD of r^2^ < 0.2. These loci were composed of 1–35 SNPs, not always adjacent. None of the significant SNPs or independent loci detected for one trait was found to be significant for another trait. The MAF of the significant SNPs ranged from 2.5% to 49.4% and averaged 36.1% for FL-*As*, 11.7% for CG-*As* and 27.5% for Ratio. The contribution of individual significant SNPs to the total variance of the trait considered (marker R2) was low and did not exceed 12%. Among the 41 SNPs significantly associated with FL-*As*, 11 corresponding to three independent loci had marker R2 *>* 10%. The highest marker R2 observed among the 23 SNPs significantly associated with CG-*As*, was 8%. Among the 82 SNPs significantly associated with Ratio, nine corresponding to six independent loci had marker R2 > 10%.

#### Association analysis in the validation population

Results of association analysis for the three traits in the VP are presented in Fig 4 and S6 Table. The number of significant associations was 15 for FL-*As*, 75 for CG-*As* and 8 for Ratio. These associations represented 8, 30 and 5 independent loci. These loci were composed of 1–22 not always adjacent SNPs, with p-values ranging between 1e-05 and 1e-09. Similar to what was observed in association analysis in the RP, in the VP significant SNP loci for the three traits did not colocalize. The MAF of the significant SNP ranged from 2.6% to 46.8% and averaged 28.0% for FL-*As*, 9.0% for CG-*As* and 9.1% for Ratio. The significant SNPs contributed much more, on average, to trait total variance than the ones observed in the RP. The mean marker R2 was 18% for SNPs associated with FL-*As*, 24% for SNPs associated with CG-*As* and 16% for SNPs associated with Ratio.

#### Congruence between the results of GWAS in RP and in VP

Among the 146 SNPs significantly associated with one of the three traits in the RP, only eight were also significant in the VP. These SNPs corresponded to one independent locus associated with CG-*As*. The application of a margin of tolerance of 1.7 Mb between a significant locus in the RP and its counterpart in the VP (corresponding to the average distances for LD of 0.2 in the VP) increased slightly the number of colocalizations: four additional colocalizations for CG-*As* and one for Ratio. The number of such colocalizations increased markedly (9, 20 and 12 for FL-*As*, CG-*As* and Ratio, respectively) when the threshold of significance of association in the two populations was lowered to a p-value < 1e-04 (Fig 4). The latter features represented 69%, 40% and 52% of the independent significant loci detected in RP for FL-*As*, CG-*As* and Ratio, respectively.

#### Genomic localization and co-localization with QTLs and gene reported in the literature

Out of a total of 146 SNPs significantly associated with one of the three *As* related traits in the RP, 41% were located in intergenic regions, 14% in introns, 27% in exons with synonymous coding effects, 10% in exons with non-synonymous coding effects, 6% in UTR-3 regions and 2% in stop-gained sites (S7 Table). The proportions were similar for the 96 significant loci in the VP and for those observed among all the 22,370 SNPs used for GWAS. Genes underlying the significant loci included ATP binding cassette involved in *As* detoxification (e.g. Os04g0620000), transporters (e.g. phosphate, ammonium, peptide, efflux transporters MATE), abiotic stress responsive genes (e.g. several F-box and DUF domain containing proteins, cytochrome P450), and transcription factors (e.g. MBY, zinc finger family protein, ERF).

A genome survey within an interval of 400 kb (200 kb downstream and 200 kb upstream) surrounding each significant SNP in the RP and in the VP, led to the identification of at least one gene with the product involved in plant response to abiotic stresses or reported in the literature as responsive to *As* stress (Fig 4 and S7 Table). The latter included OsLsi1, OsHAC1, OsHAC6, OsACR2-1 and representative of glutathione S-transferases, Cytochrome P450s, heat shock proteins, metal-binding proteins, phosphate acquisition proteins, transporter proteins and transcription factors. Likewise, survey of the surrounding interval of 400 kb of the significant SNPs for QTL reported in the literature to be associated with *As* resulted in a large number of colocalizations (Fig 4 and S7 Table).

### Genomic prediction

#### Cross-validation experiment in the reference population

The nine cross-validation experiments involving the three prediction methods and the three phenotypic traits yielded predictive ability ranging from 0.326 to 0.543 (Table 2). The average predictive ability was 0.452 for FL-*As*, 0.535 for CG-*As* and 0.333 for Ratio. Based on ANOVA results, differences in predictive ability between the three traits were highly significant (P < 0.0001). Conversely, differences in predictive ability between the three prediction methods were not significant. This was also the case for interactions between prediction methods and phenotypic traits.

**Table 2:** Predictive ability (r) of three methods of genomic prediction for three rice arsenic content traits in the reference population, based on cross-validation experiments.

Prediction method	Phenotypic traits						
	FL-*As*		CG-*As*		Ratio		
	r	sd	r	sd	r	sd	Average r
BayesA	0.447	0.095	0.530	0.118	0.326	0.130	**0.435**
GBLUP	0.450	0.097	0.531	0.097	0.328	0.132	**0.437**
RKHS	0.457	0.094	0.543	0.123	0.343	0.130	**0.448**
**Average**	**0.452**	0.095	**0.535**	0.113	**0.333**	0.131	**0.440**

FL-*As*: flag leaf arsenic content; CG-*As*: cargo grain arsenic content; Ratio: CG-*As*/FL-*As*. r: average predictive ability; sd: standard deviation.

In order to evaluate the effect of exclusion of highly redundant SNP (r^2^ = 1), the cross-validation experiment was also implemented with the full set of SNPs available (22,370), under GBLUP. Results showed negligible effects on predictive ability: r = 0.449 versus 0.450 with the incidence matrix of 16,902 for FL-*As*, r = 0.535 versus 0.531 for CG-*As*, and r = 0.326 versus 0.328 for Ratio.

#### Genomic prediction across populations

**Fig 5:**
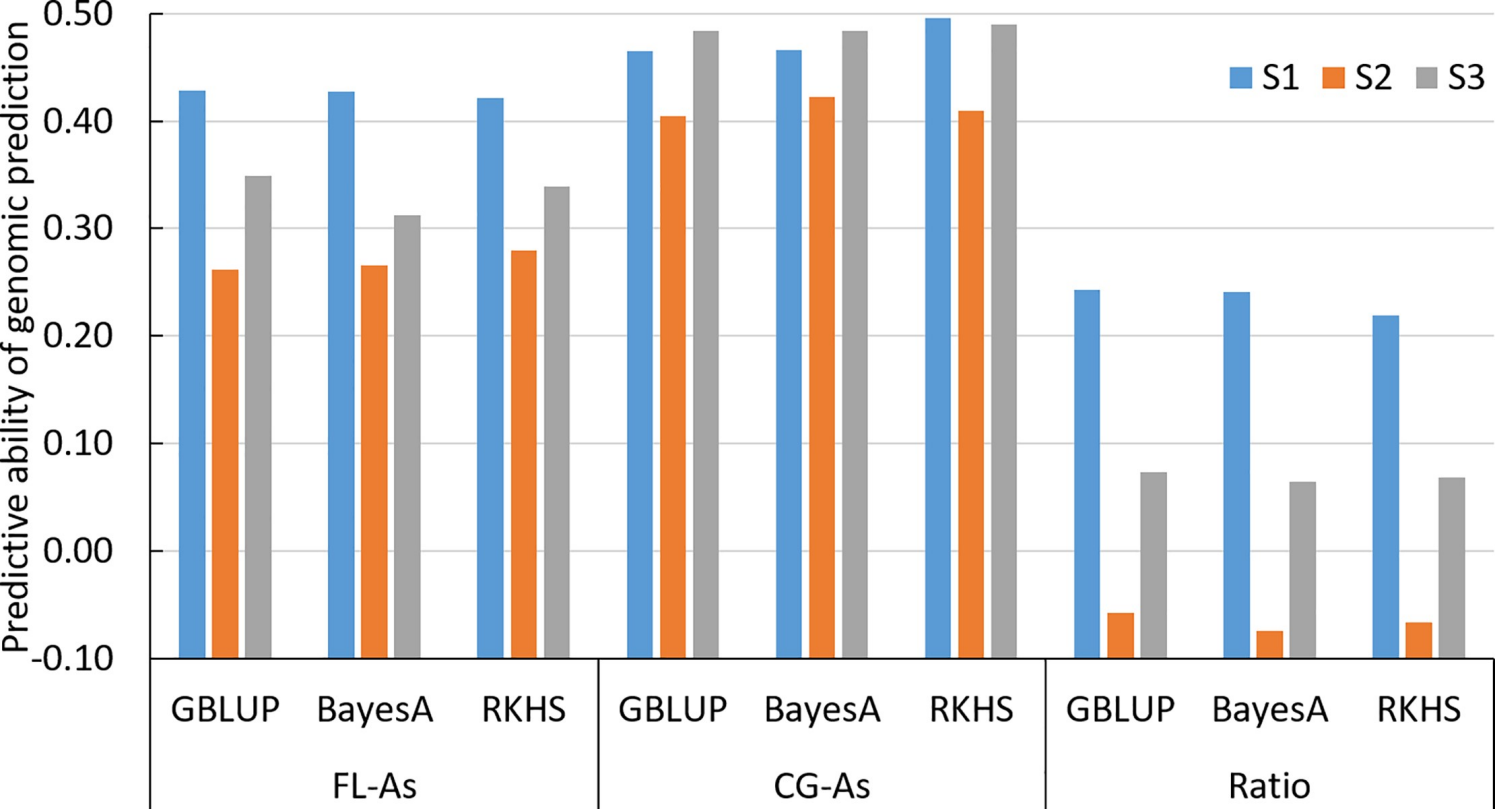
Predictive ability of genomic prediction of the arsenic concentration in the flag leaf (FL-*As*) in the cargo grain (CG-*As*), and for the CG-As/FL-*As* ratio of the validation population obtained with three statistical methods, BayesA, GBLUP and RKHS, under three scenarios of composition of the training set.

Under the S1 scenario, using all the 228 accessions of the RP as the training set, the predictive ability of genomic estimate of breeding value (GEBV) of the 95 lines of VP was on average 0.426 for FL-*As*, 0.476 for CG-*As* and 0.234 for Ratio (Fig 5 and S8 Table). The three prediction methods implemented provided similar levels of predictive ability. However, there was some interaction between prediction methods and phenotypic traits. Similar to the cross-validation experiments, the addition of the redundant SNPs in the incidence matrix did not noticeably modify the predictive ability (Fig 5).

The predictive ability of GEBV were much lower under S2, with averages of 0.266, 0.411 and -0.016 for FL-*As*, CG-*As* and Ratio, respectively (Fig 5). Under S3, the average predictive ability was slightly higher than under S1 for CG-*As* (0.491), and much lower than under S1 for FL-*As* (0.341) and for Ratio (0.073).

## Discussion

The aim of this work was to explore (i) the phenotypic diversity of the rice *japonica* subspecies, adapted to cultivation in Mediterranean Europe, to restrict *As* accumulation in the edible grains, and (ii) the potential of the two major options of marker-assisted selection for the improvement of the trait, i.e. QTL-based selection and genomic estimate of breeding value (GEBV)-based selection.

### Phenotypic diversity for arsenic content

Phenotypic diversity for *As* accumulation in the flag leaf and in the cargo grain was evaluated in field experiments with uncontrolled intensity of exposure to *As*. However, a rather stable soil *As* concentration, of about 10 mg kg^-1^, was observed across the crop cycles, and in the three consecutive years of field experiments. This concentration corresponded to the class of rather high *As* contents reported for paddy fields in countries including Bangladesh [66], China [67] and the USA [66]. The range of variation of CG-*As* (0.147 to 0.656 mg kg^-1^) among the accessions of RP was similar to the range observed by [29, 32] in a panel of some 400 accessions representative of the diversity of all the *O*. *sativa* species (http://www.ricediversity.org/), evaluated during multilocation trials in Bangladesh, China and USA. Relationship between FL-*As* and CG-*As* was rather loose suggesting there are differences between accessions in the ability to limit *As* transfer from the leaves to the grains. To our knowledge, the existence of such genetic diversity for the CG-*As*/FL-*As* ratio has not been reported yet in the literature. Correlation between the FL-*As* and CG-*As* observed in 2014 and 2015 for the 50 RP accessions of RP that were evaluated in the two consecutive years was rather high. This confirmed the robustness of our findings concerning the extent of genetic diversity for FL-*As* and CG-*As* and on the relationship between the two traits. Interestingly, the extent of FL-*As* and CG-*As* in the VP was as large as that observed in the RP, despite its much smaller size.

### Detection power and robustness of GWAS

In order to explore the potential of marker-QTL association-based breeding for aptitude to restrict *As* accumulation in the grains, association analysis was performed in the RP to detect QTLs. A large number of QTLs was detected for each of the three traits considered. Some of these QTLs colocalized with already reported QTLs [27–28], candidate genes [21, 67], or cloned genes [21, 37]. To ascertain further the results of GWAS in the RP a validation experiment was performed in an independent population, our VP, as recommended in the literature [68, 69]. GWAS with the VP also detected a large number of SNP and independent loci. However, only a few QTLs detected in the VP colocalized with the QTLs detected in the RP, despite loosening of the interval surrounding each QTL, or lowering the significance threshold from 1e-05 to 1e-04. Replication of GWAS experiment requires similarity for several features between the two populations and sufficiently large validation population to ensure detection power [70]. Our validation experiment fulfilled all similarity conditions as VP had similar population structure to RP (composed of temperate and tropical *japonica*), similar relationship between population structure and variability of the target trait (the temperate *japonica* having the highest *As* contents) and similar MAF distribution. Thus, the limited colocalisation between the QTL detected in RP and in VP should be attributed mainly to the rather small size of the VP.

Given the above-mentioned superposition of the distributions of the phenotypic variability and the structuring of RP and VP into temperate and tropical *japonica*, our GWAS results might have been subject to an abnormal rate of false negatives due to a confounding phenomenon [71]. To evaluate this risk, separate association analyses were performed with the 153 temperate and the 75 tropical *japonica* accessions of RP. These analyses detected, at best, 50% of the QTLs detected with the entire RP, without markedly increasing the P-value for each association (S9 Table). The expected positive effects of diverting the confounding phenomenon proved to be smaller than the reduced detection power due to the reduced size of the population.

The conclusions drawn from these results are that (i) it is unlikely that a single GWAS makes it possible to establish robust and precise genotype–phenotype associations, especially for complex traits, (ii) validation of GWAS results by a second independent GWAS experiment is a complex process with uncertain results, (iii) new validation options, such as development of b-parental mapping population or the genome editing approach [72], need to be explored. Some of the QTLs our two GWAS experiments have identified colocalized tightly enough with candidate genes to deserve such validation effort. This is the case, for instance, for qAs-01 (Os01g74300, Metal-Binding Proteins), qAs-07 (Os07g12130, MYB family transcription factor), qAs-08 (Os08g16260, cytochrome P450 protein), qAs-10 (Os10 g38600, Glutathione S-transferases) and qAs-12 (Os12g36670, F-box/LRR-repeat protein involved in response to abiotic stresses).

### Predictive ability of genomic predictions

To explore the potential of genomic prediction options for marker-assisted breeding for the ability to restrict *As* accumulation in the grains, three prediction methods were tested. Although these methods represented contrasted hypotheses regarding the effects of markers and/or the genetic determinism of target trait (see M&M section), they did not show significantly different predictive ability for FL-*As* and CG-*As*. This is in agreement with the large number of QTLs of rather small effect detected in our GWAS experiment and suggest a mainly additive genetic determinism of FL-*As* and CG-*As*. Similar limited differences in predictive ability between prediction methods, for complex traits, were reported in rice [51, 52] and in other crops [73, 74]. Given these limited differences, the RKHS method which can capture both additive and more complex genetic effects seems to be the most recommendable option. The exclusion of the most redundant SNP markers, based on LD information, had a limited effect on predictive ability, confirming the fact that accounting for LD in the population matters more than the absolute marker density [75].

The level of predictive ability for FL-*As* and CG-*As* in the cross-validation experiments was similar to the levels reported in the literature for traits of equivalent heritability in rice [52] and other major crops [74–75]. Predictions were less accurate for the Ratio trait, which, by design, accumulates the experimental noises associated with the evaluation of FL-As and CG-As, and had lower heritability.

Across population genomic prediction with models trained with RP data led to slightly lower predictive ability than the predictive ability observed in the cross-validation experiments. Similar decreases in predictive ability were reported in rice [52], barley [74] bread wheat [76], and strawberry [77] and were attributed to differences in LD and allele frequencies between the training and the validation sets [78]. In our case, no significant differences in predictive ability were found between the GBLUP model that captures marker-based relationship between RP and VP, and RKHS and BayesA that captures LD between markers and QTLs. An attempt to reduce the discrepancy in allele frequency between RP and VP by discarding SNP loci with highly divergent MAF did not markedly change predictive ability (data not shown). Neither could conclusive improvement in predictive ability be achieved by optimizing the composition of the training set using the CD-mean approach [65]. These findings suggest that further research aimed at improving the predictive ability of across population genomic predictions should explore the effects of the size of the training set (using a larger training set) and of the balance between marker density and the regularity of their distribution along the genome. Indeed, in the present work, marker density (one SNP every 17.1 kb) was rather high, given the extent of LD, but their distribution was not optimized given the GBS genotyping technology. Another option that deserves further research is the use of the available QTL information in genomic selection. These QTLs’ information could serve to build trait-specific genomic relationship matrices, based on the modified VanRaden genomic relationship matrix, with marker weights for each locus, proposed by [79].

### Implication for breeding rice to prevent *As* accumulation in the grains

The critical importance of reducing the presence of *As* in the rice grains in a large proportion of rice growing areas has recently resulted in steady efforts to understand the molecular mechanisms involved in plant response to overexposure to *As* [12, 24] and the genetic control of these mechanisms [17, 18, 21]. Although a few genes, reported as being “crucial”, have been cloned [38], transcriptome analyses [23, 67] and GWAS results [32] suggest that *As* tolerance is a complex trait involving large number of loci with limited individual effect on the trait.

The number of candidate loci makes marker-assisted pyramiding of the favorable alleles unpractical. Moreover, uncertainty concerning the exact genomic position of some of the loci makes the outcome of marker-assisted pyramiding unpredictable. Indeed, as discussed above, GWAS results raise robustness issues and this also seems to be the case for transcriptome analyses [68].

The GEBV obtained for FL-*As* and CG-*As* were reasonably accurate in both intra-population (cross- validation in the RP) and across-population (RP/VP) prediction experiments. Translation of those predictive abilities into average phenotypic performances of VP lines selected based on their GEBV, by model trained with the RP, is even more encouraging. Indeed, the average FL-*As* and CG-*As* of the best 10 VP lines selected on the basis of phenotypic data were 41% and 65% of the average FL-*As* and CG-*As* of all 95 lines of VP. The average FL-*As* and CG-*As* of the best 10 VP lines selected on the base of GEBV were 55% and 85% of the average FL-*As* and CG-*As* the all 95 lines of VP (S10 Table). In other words, for a selection rate of 10%, the difference in genetic gain between phenotypic selection and GEBV based selection was approximately 10% for FL-*As* and 5% for CG-*As*. Given these rather small differences in genetic gains, at first sight the choice between phenotypic and GEBV based selection will depend mainly on the comparative costs of genotyping and phenotyping for *As* content. If the costs are similar, the best choice would be GEBV-based selection because genotypic data are a multi-purpose asset that can also be used for genomic prediction of other traits than *As* content. However, integration of GS in a breeding program has multiple implications [80, 81] that require thorough evaluation.

To conclude, considering the limitations of QTL-based marker-assisted selection for *As* and the rather high level of predictive ability of GEBV, the hypothesis that genomic prediction offers the most effective marker assisted breeding approach for the ability to prevent *As* accumulation in the rice grains, proved to be exact. This is especially the case for breeding programs that have already adopted GS approach for other traits. It was shown that a rice diversity panel could provide accurate genomic predictions for complex traits in the progenies of biparental crosses involving members of the panel [52]. In addition, associated with the rapid generation advancement technique, genomic selection can accelerate the genetic gain of the pedigree breeding scheme, the most common breeding scheme in rice. GS for *As* content can be incorporated rather easily in such breeding programs. The main additional cost would be the phenotyping of the diversity/reference panel for *As* content.

## Supporting information

S1 Table Main characteristics of the 300 accessions of the reference population (RP) and 95 advanced lines of the validation population. (XLSX)

S2 Table Soil and water arsenic contents in the experimental site over the three years of field experiments. (XLSX)

S3 Table Genotypic data (22,370 SNP markers) for 228 accessions of the reference population and 95 advanced of the validation population in HapMap format. (ZIP)

S4 Table Variability of marker density and frequency of minor alleles (MAF) along the 12 chromosomes in the reference and the validation populations. (XLSX)

S5 Table Average arsenic contents of the two subgroups of *O*. *sativa japonica* present in the reference population (RP) and in the validation population (VP). (XLSX)

S6 Table Results of association analysis of the concentration of arsenic in the flag leaf (FL-As) in the cargo grain (CG-As), and for the CG-As/FL-As ratio, in the reference population (RP) and in the validation population (RV). (XLSX)

S7 Table Colocalization of SNP loci significantly associated with arsenic content traits in the present study with similar loci reported in the literature. (XLSX)

S8 Table Predictive ability of genomic estimate of breeding value of the 95 advanced lines of the validation population for arsenic contents, by three genomic prediction models trained with data from 228 accessions of the reference population. (XLSX)

S9 Table Results of association analysis of the concentration of arsenic in the flag leaf (FL-As) in the cargo grain (CG-As), and for the CG-As/FL-As ratio, in the temperate *japonica* component (175 accessions) of the reference population (RP). (XLSX)

S10 Table Translation of predictive ability of genomic prediction into genetic gain under different selection intensities. (XLSX)

S1 Fig Distribution of the 22,370 working set SNP markers along the 12 chromosomes in the reference and validation populations. (TIF)

S2 Fig Distribution of adjusted phenotypic values for arsenic content of the flag leaf (FL-As) and arsenic content of the cargo grain (CG-As), in the reference and validation populations, according to membership of the accessions of temperate japonica and tropical japonica subgroups. (TIF)
