# Supplementary figures and images for: Genomic prediction offers the most effective marker assisted breeding approach for ability to prevent arsenic accumulation in rice grains
Source: PLoS One. 2019 Jun 13;14(6):e0217516. doi: 10.1371/journal.pone.0217516 (PMC6563978; doi:10.1371/journal.pone.0217516)

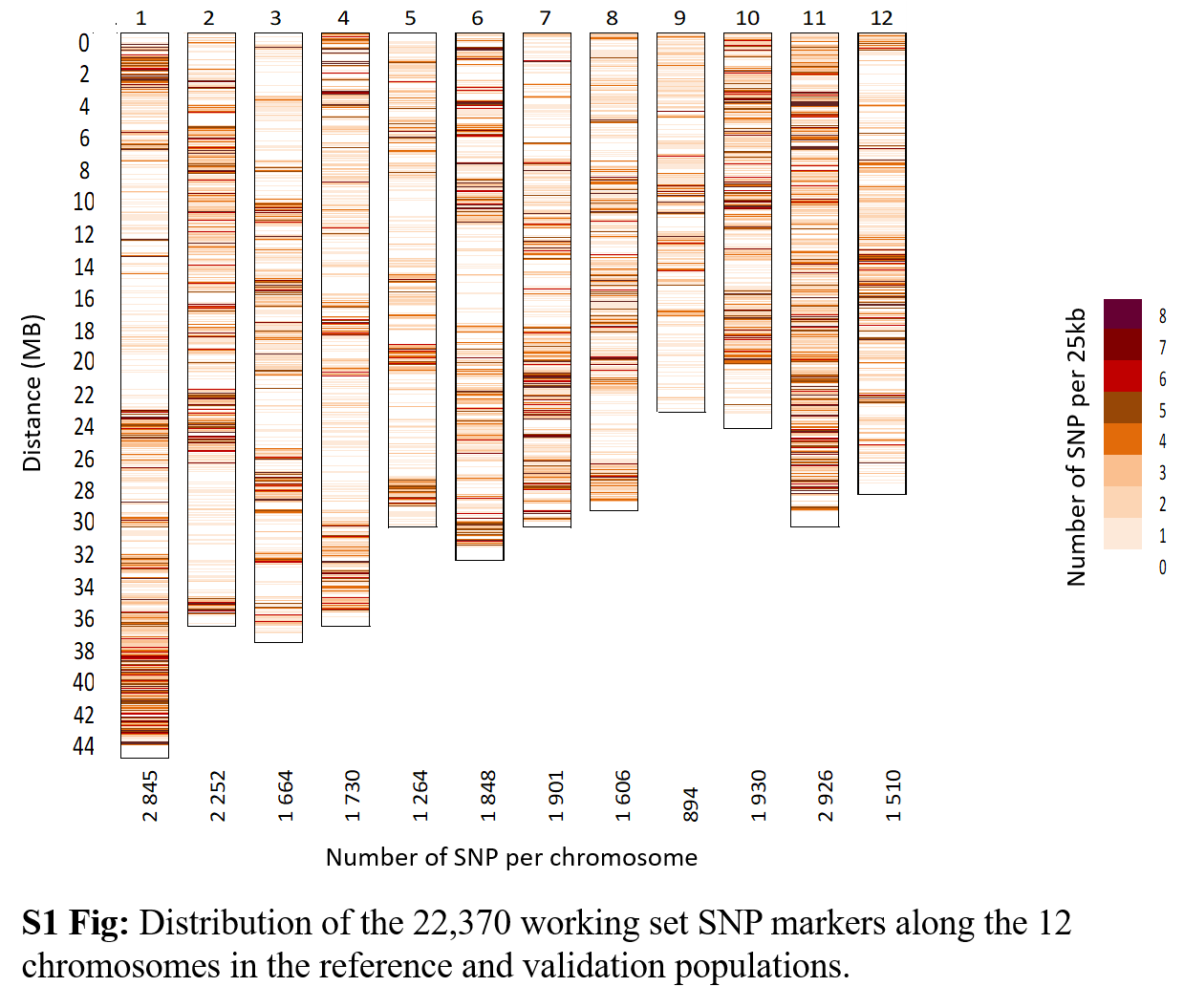

Supplement: S1 Fig — (TIF) [file pone.0217516.s011.tif]

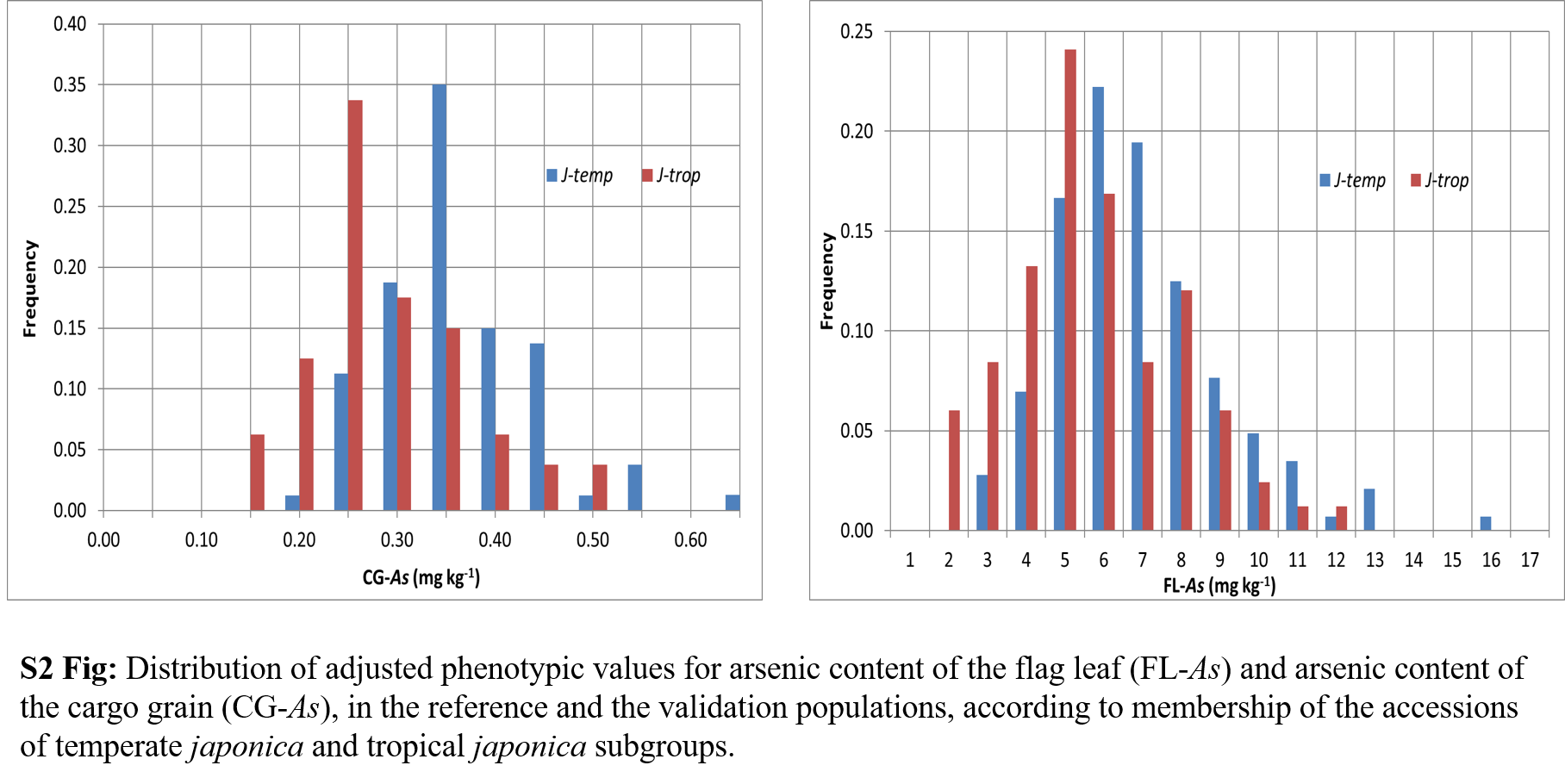

Supplement: S2 Fig — (TIF) [file pone.0217516.s012.tif]
